# The river meeting the sea: A qualitative exploration of the healthcare transition experiences of adolescents and young adults living with rare renal disorders and their parents

**DOI:** 10.1371/journal.pone.0348445

**Published:** 2026-06-03

**Authors:** Melissa Kinch, Thilo Kroll, Suja Somanadhan

**Affiliations:** 1 School of Nursing, Midwifery & Health Systems, University College Dublin, Ireland; 2 University College Dublin Centre for Interdisciplinary Research, Education & Innovation in Health Systems (IRIS Centre), Ireland; University of Ibadan, NIGERIA

## Abstract

**Background:**

Rare renal disorders are a group of complex conditions that can lead to progressive kidney failure and lifelong multi-system complications. Upon reaching young adulthood, adolescents and young adults must navigate the healthcare transition between paediatric and adult services. This process serves to bridge the gap between health services and provide adolescents and young adults with developmentally appropriate support to manage adult life with their condition. However, this process can prove challenging for adolescents and young adults with rare renal disorders, a research area that is currently under explored.

**Aim:**

To explore the experiences of adolescents and young adults and parents living with a rare renal disorder and undergoing healthcare transition.

**Design:**

Qualitative descriptive study, using reflexive thematic analysis. Results reported according to the COnsolidated Criteria for REporting Qualitative research (COREQ) checklist.

**Methods:**

Twenty eight in-depth interviews were conducted, with 17 parents and 11 adolescents and young adults with rare renal disorders.

**Results:**

Five themes were developed: 1. the complex and ever-changing nature of rare renal disorders, 2. preparing to move on, 3. understanding the person, 4. building support networks, and 5. care coordination, consistency, and communication. Adolescents and young adults and their parents recognised the need for individualised, collaborative, and holistic approaches to healthcare transition, emphasising the need for comprehensive support that acknowledges other areas of adolescents and young adults lives, including educational transitions, peer connection and psychological support.

**Conclusion:**

This study emphasises the dynamic interplay between health and social systems when planning healthcare transition. This study offers valuable insights into healthcare transition in rare renal disorders. Findings provide a foundation for future research and can inform practice, policy and the development of future healthcare transition interventions.

**Patient or Public Contribution:**

Two adolescents and one parent from a rare-disease advisory group provided input on the study materials.

## 1. Introduction

In Europe, Rare Renal Disorders (RRDs) encompass more than 300 conditions and nearly all paediatric kidney disorders [1]. Despite medical, technological and scientific advancements in genetic testing, diagnostics and drug development, Adolescents and Young Adults (AYAs) with RRDs face challenges throughout life, largely due to condition heterogeneity, multi-system complexities, fragmented care and subpar genomic literacy [2–5]. These AYAs often experience physical and psychosocial challenges. For example, they may undergo aggressive treatment with side effects, such as Renal Replacement Therapy (RRT). Renal replacement therapies, while transformative in improving AYA survival and long-term health prospects, can carry a considerable physiological and psychological burden [6]. Likewise, from a social perspective, AYAs can miss time from school and face social exclusion from activities with peers, leading to isolation and stigma [7,8]. These challenges are exacerbated during adolescence, a period marked by rapid physical, emotional, and psychosocial change [9].

Adolescents and young adults with RRDs experience pivotal life transitions during adolescence, including significant educational changes such as transitioning between school stages or entering higher level educational institutions. Adapting to these transitions can be challenging and is often accompanied by uncertainty and increased responsibility [10]. Adolescents and young adults must navigate these life transitions amid unique health transitions, such as the Healthcare Transition (HCT) from paediatric to adult health services. The HCT is formally known as the ‘purposeful, planned process that addresses the medical, psychosocial, educational and vocational needs of adolescents and young adults....as they grow up learning to live with their lifelong health condition’ (Dovey-Pearce and Christie, 2013, p.175) [11]. This process encompasses preparation in paediatric services, transfer to adult providers, and supported acclimatisation to adult services [5,12].

While most life transitions, such as puberty, follow variable timelines for AYAs, HCT often follows rigid time frames and norms grounded in health service considerations. During early adolescence, it is recommended that AYAs commence HCT preparation [13]. The transfer event generally occurs during late adolescence (16–18 years) or emerging adulthood (18–25 years) [5,12]. However, not all AYAs may be ready to transfer to an adult health service environment for a variety of reasons, including health-related concerns (for example, worry about losing paediatric practitioners with specialist expertise), personal fears, and competing developmental challenges (for example, concurrent educational changes). If the disconnect between transition readiness and service requirements is not recognised, consequences can include poorly managed transitions and adverse health outcomes, such as graft loss for transplant recipients, at an already precarious time [14,15].

Globally, AYAs have the highest rates of kidney graft loss among all groups [16,17]. The reasons for this are multifactorial and can include psychosocial factors (for example, treatment non-adherence) and biological factors (for example, increased immunological or metabolic activity) [18,19]. From a social perspective, condition heterogeneity may culminate in isolating experiences and fewer support networks [20,21]. From the health service perspective, the low prevalence and complexity of RRDs mean that adult specialists with the requisite expertise are relatively few, typically located in specialised tertiary or reference centres [1]. These circumstances emphasise the need for tailored HCT programmes. To date, HCT in the context of RRDs has been largely unexplored both nationally and internationally. This study serves to bridge this gap. This study explores the life and HCT experiences of AYAs living with RRDs and their parents.

The following research question was posed:

‘How do adolescents and young adults living with rare renal disorders and their parents experience healthcare transition?’

This study had two distinct, yet interconnected objectives. This paper first sought to understand how AYAs and parents experienced life with an RRD. This contextual information was considered to be important because it provided insight into the additional layers of complexity that AYAs and parents experience every day, which may affect HCT or their ability to cope with or navigate this process. The second, and primary, aim of this study was to explore AYAs’ and parents’ HCT-specific experiences.

## 2. Materials & methods

A qualitative descriptive approach was employed, using semi-structured interviews. Qualitative descriptive studies provide straightforward descriptions of participants’ experiences [22]. This approach can be particularly useful when exploring poorly understood topics, such as HCT in RRDs [22,23]. The approach recognises the vast and differing perspectives that participants may have [22]. It was considered particularly useful for this study, where the goal was to provide direct accounts of the HCT process while remaining close to participants’ own words [23]. This study was reported according to the COnsolidated Criteria for REporting Qualitative Research (COREQ) checklist [24] (see S1 File for completed checklist).

### Ethical Considerations

The study received ethics approval from the relevant ethics boards of a paediatric health service research and academic institution (LS-24–32-Kinch-Somanadhan and REC-384–24). Participants provided informed written consent after receiving information about the study through participant information leaflets. For minors under 18 years, parents provide written, informed consent, and minors provide written, informed assent. Participant information leaflets included information about the voluntary nature of participation, secure storage and protection of data, confidentiality and the right to withdraw from the study up to irreversible data anonymisation. Additional information on participant recruitment is available in the sampling and recruitment section below.

### Patient and Public Involvement

Patient and Public Involvement (PPI) involves working collaboratively with patients and the public in research [25]. Stakeholder participation in research can vary from consultation to partnership [26]. The researchers consulted with established PPI groups during the design of this study. Two AYAs and a parent from national rare disease advisory groups were consulted to advise on study materials. Additionally, a child health information specialist reviewed and supported the development of the study materials for AYAs under 18 years.

### Sampling & Recruitment Procedures

This study applied a maximum variation sampling strategy, given the recognised heterogeneity of RRDs [27]. Maximum variation sampling was used to recruit participants who had a wide range of perspectives and experiences relevant to the topic [27]. Participants included AYAs and parents attending paediatric services, as well as those who had transitioned to adult services across the Republic of Ireland. The AYAs were eligible to participate if they 1. were between twelve and twenty-four years old, 2. were living with an RRD, 3. were receiving treatment in the Republic of Ireland, and 4. could communicate in English. The sampling strategy was selected in recognition of transition recommendations which suggest that young people and their families should commence transition preparation early and that the transition process does not conclude with the transfer event [12,13].

Parents were eligible if 1. they were a parent who was currently supporting or had supported a child living with an RRD with HCT in the past, 2. their young person was receiving treatment in the RoI, and 3. they could communicate in English. Participants were ineligible if they 1. were experiencing an illness that impacted their ability to participate, 2. had significant cognitive impairment that impacted their ability to consent or assent to the study or understand the interview questions or procedures (per clinician or parental advice), or 3. received a transplant of commenced dialysis less than six months previously.

This can be a challenging recovery period for AYAs and their families. This criterion reduced the risk of avoidable distress among prospective participants. Table 1 presents the inclusion and exclusion criteria.

**Table 1:** Inclusion/Exclusion criteria for adolescents and young adults & parents.

Inclusion & Exclusion Criteria (Adolescents and Young Adults)	
**Inclusion Criteria**	**Exclusion Criteria**
Adolescent and young adults aged 12–24 years who have experienced or are experiencing the movement between paediatric and adult health services.	Adolescents and young adults who were experiencing acute illness at the time of the study and who were unable to participate.
Adolescents and young adults living with a rare renal disorder.	Adolescents and young adults who have significant cognitive impairment impacting their ability to consent or assent to the research or understand the study procedures (per parental or clinician guidance).
Adolescents and young adults who are receiving treatment in the Republic of Ireland.	Adolescents and young adults who had received a transplant or commenced dialysis less than six months previously.
Adolescents and young adults who can communicate in English.	
**Inclusion & Exclusion Criteria (Parents)**	
**Inclusion Criteria**	**Exclusion Criteria**
Parents of young people with a rare renal disorder.	Parents of young people who had received a kidney transplant less than six months previous.
Parents who are currently supporting or have supported a child with healthcare transition in the past.	
Parents of young people receiving treatment in the Republic of Ireland.	
Parents who can communicate in English.	

Multiple recruitment strategies were employed to account for the heterogeneity of the sample. Participants were recruited through social media, clinics *via* a gatekeeper, and advocacy groups using flyers. Interested participants contacted the researchers directly by phone or email. For minors under 18 years, parents made contact with the researchers. The researchers issued information leaflets, consent and assent forms to prospective participants. Pre-interview screening was conducted *via* Zoom, phone or email to confirm eligibility, address questions, establish rapport and describe the research. Participation remained entirely voluntary. All participants were offered up to two weeks to consider participation before providing consent. All participants provided informed, written consent to participate. For minors under 18 years, parents provided informed, written consent, and the young people provided informed, written assent.

Sampling and recruitment procedures continued until sufficient information power was reached [28], which was established when conducting additional interviews yielded no novel insights. Braun and Clarke [29] acknowledge that there are other ‘pragmatic’ considerations that may influence the researcher’s decision or judgment surrounding when to discontinue data collection. For example, when the researcher reaches the desired sample diversity or pragmatic constraints, such as time or funding [29]. Therefore, other factors that influenced the final sample size included: 1. the basis that the research team had reached a consensus that the data were sufficient to meet the research aims and address the research question, 2. diverse perspectives had been represented through maximum variation sampling, for example, the voices of AYAs and parents with different conditions and of various ages, and 3. a team-based evaluation of the value of continuing recruitment and collecting new data, given that the recruitment period lasted a protracted period of approximately nine months, and all recruitment channels had been pursued [29]. The researchers were cognisant of the resources available, including limited time to complete this study as part of the primary author’s academic degree and finite financial resources.

### Participants

A total of 28 participants self-selected to take part in the study (17 parents and 11 AYAs). Of these, (n = 18) were individual, in-depth interviews with parents and AYAs from the same family, and (n = 2) were individual, in-depth interviews with two parents (mother and father) from the same family. To uphold participant anonymity, potentially identifiable information has been redacted from this manuscript. In addition, participants’ conditions and gender are not reported in aggregate. This measure was considered important to minimise the risk of deductive disclosure in this heterogeneous sample, given the small number of AYAs living with RRDs and experiencing HCT in Ireland each year [30].

The researcher sought maximum diversity in conditions. Conditions represented included Nephrotic Syndrome, Tuberous Sclerosis Complex, Cystinosis, and diverse Congenital Anomalies of the Kidney and Urinary Tract (CAKUT). There was also diversity in treatment modalities, with kidney transplant recipients, dialysis patients, and patients receiving alternative therapies represented. Sample demographics are presented in Table 2. The higher parental representation may be attributed to parents of AYAs who could not contribute to the study due to illness, parents sharing their experiences on behalf of an AYA with cognitive impairment or intellectual disability, and AYAs who did not wish to participate in the study. The challenges of recruiting AYAs have long been reported in the literature [31–33].

**Table 2:** Sample demographics.

Young People		
**Participant Code**	**Stage of Transfer**	**Age**
**AYA-1**	Preparing	12 years
**AYA-2**	Preparing	15 years
**AYA-3**	Preparing	15 years
**AYA-4**	Completed HCT	20 years
**AYA-5**	Preparing	15 years
**AYA-6**	Preparing	13 years
**AYA-7**	Preparing	15 years
**AYA-8**	Completed HCT	22 years
**AYA-9**	Completed HCT	16 years
**AYA-10**	Preparing	17 years
**AYA-11**	Completed HCT	24 years
**Parents**		
**Participant Code**	**Stage of Transfer**	**Mother/Father**
**P-1**	Completed HCT	Mother
**P-2**	Completed HCT	Mother
**P-3**	Preparing	Mother
**P-4**	Preparing	Mother
**P-5**	Preparing	Mother
**P-6**	Preparing	Mother
**P-7**	Completed HCT	Mother
**P-8**	Preparing	Mother
**P-9**	Completed HCT	Mother
**P-10**	Completed HCT	Mother
**P-11**	Preparing	Mother
**P-12**	Preparing	Mother
**P-13**	Completed HCT	Mother
**P-14**	Completed HCT	Mother
**P-15**	Completed HCT	Father
**P-16**	Preparing	Mother
**P-17**	Completed HCT	Mother

Potential barriers to AYA involvement encountered in this study, as described by AYAs and parents, included insufficient time to participate due to competing demands (for example, school or sports), reluctance to engage in medical-related discussions outside of hospital appointments, and lack of interest in participating in the research. The researchers were aware that greater parental representation could pose challenges, with a potential for parents’ voices to dominate the results. To address this challenge, AYA and parental data were continuously compared throughout the analysis, as discussed in more detail in the data analysis section below.

### Interview process

Semi-structured interviews were conducted between June 2024 and March 2025. The primary researcher, a female paediatric nurse and doctoral student, trained in qualitative methods and interviewing techniques, conducted the interviews. Interview guides were informed by the HCT framework of Betz et al. [34], the National Model of Care for Rare Diseases [35] and a previous scoping review conducted by the lead author [5] (see S2 File for interview guides). The first parent and AYA interviews served as pilot interviews. The interview guide required minimal revisions following these pilots. These revisions primarily involved wording changes to improve participants’ comprehension of the questions. Given that the amendments to the interview guide were minimal, the pilot interviews were included in the final dataset. Given the semi-structured nature of the interviews, they did not always follow a predefined format. Instead, the researcher allowed the participants’ perspectives and experiences to guide the discussions.

Participants had the option of in-person or Zoom interviews. Four participants opted for in-person interviews, which were conducted at the paediatric outpatient department. The remainder were conducted online *via* Zoom. Participants under 18 years had the option to have a parent present during the interview. The literature is inconclusive about the benefits of parental presence [36].

On the one hand, AYAs may be less likely to open up or may become reliant on parents when they are present [36]. Conversely, parents may support AYAs comfort and participation [36]. Cognisant of these considerations, participants under 18 years had the option to have a parent present. Four participants opted to have their parents remain present for their interviews. The average interview length was 46 minutes for parents and 38 minutes for AYAs. While the researchers can only speculate about the reason for the shorter interviews for the young people, the duration may have reflected differences in experiences and conversational style [37,38]. The younger participants often offered more concise responses to the interview questions, potentially reflecting developmental factors, such as their experience engaging in reflective discussion and expressing their thoughts and perspectives [37,38]. For example, Heath et al. [37] explained how some young people may be more attuned, feel more comfortable or be more able to articulate their views than others.

After the interview, participants were invited to provide additional comments if they wished to elaborate on any aspect of HCT or discuss a topic that was not covered in the interview guide. Researchers audio-recorded and self-transcribed the interviews verbatim, then uploaded the anonymous transcripts to an encrypted drive accessible only to the researchers. Identifiable consent and assent forms were stored separately from the interview transcripts. Audio recordings were irreversibly destroyed once transcribed.

### Data Analysis

Data were analysed inductively using Reflexive Thematic analysis (RTA), a qualitative analysis approach for developing, analysing, and interpreting themes, comprising six iterative steps: 1. Data familiarisation, 2. Coding, 3. Candidate theme development, 4. Theme development, 5. Refining, defining and naming themes, and 6. Writing up [39]. Transcripts were uploaded to NVivo 2020 (NVivo Release 1) for data management and analysis.

The researchers familiarised themselves with the data by reading and making reflexive notes. Thereafter, the first author conducted line-by-line coding with support from the third author. Both authors met regularly throughout this process to discuss codes and conflicts. The whole team also met regularly to support the transition from code to category and themes or sub-themes. Themes and sub-themes were then developed and refined. The themes selected were grounded in and reflected the participants’ perspectives and experiences [22,23]. Each theme was supported by verbatim quotes. Table 3 provides the coding tree followed by the researchers during data analysis.

**Table 3:** Coding tree.

Theme	Sub-themes	Categories	Sample Codes	Theme Descriptor
**Theme 1:** The Complex and Ever-Changing Nature of a Rare Renal Disorder	**Sub-theme 1.1:** An uncertain future	Anticipating uncertainty	-Rollercoaster -Coming to terms with the diagnosis -Diagnostic odyssey	The ever-changing nature of a rare renal disorder is highlighted, along with challenges in receiving a diagnosis. This theme describes the everyday challenges faced by young people with rare renal disorders. This includes the burden on their daily life, impact on family life, including siblings, and the multi-system nature of these challenging conditions.
		Changing condition	-Changing needs when living with a rare renal disorder -Changing condition	
	**Sub-theme 1.2:** The toll of Rare Renal Disorders on everyday life	Living under the weight of an RRD	-Emotional toll of a rare renal disorder -Simultaneous life transitions	
		Impact of a rare renal disorder on daily life	-Burden of disease on everyday activities -Impact on family life -Impact on siblings	
		Multi-faceted condition	-Complex needs or intellectual disability -Multisystemic nature of rare renal disorders	
**Theme 2:** Preparing to Move On & Managing Change	**Sub-theme 2.1:** Age and Healthcare Transition Readiness	Tailoring developmentally appropriate support	-Developmental level -Age -Letting go -Readiness to transition	This theme describes the need to support young people in understanding their condition and managing it appropriately. This theme emphasises how young people navigate healthcare transition.
	**Sub-theme 2.2:** Empowering Adolescents and Young Adults Throughout Healthcare Transition	Empowering young people to understand their condition and own their care.	-Self-advocacy or management -Decision-making abilities	
	**Sub-theme 2.3:** Navigating change and disrupted expectations	Empowering young people and families to navigate Healthcare Transition	-Perceived level of preparation -Comparing the paediatric setting with the adult setting	
**Theme 3:** Understanding the Person and Providing Holistic, Individualised Support	**Sub-theme 3.1:** Prioritising mental health	Challenging emotions and psychological sequelae	-Anxiety -Isolation -Uncertainty -Guilt	This theme highlights the psychological impact of rare renal disorders and how this impact is heightened during life transitions such as healthcare transitions.
		Need for psychological support	-Practical help -Falling through the cracks -Gaps in mental health care -mental health support	
	**Sub-theme 3.2:** Two sides of the same coin: Integrating life and Healthcare Transition	Educational or vocational support	-In hospital education support -Educational support outside of hospital -Role of school teachers and other staff, e.g., guidance counsellors	This theme emphasises the need to consider the young person’s educational and vocational needs when delivering healthcare transition support.
		Positive and negative experiences in school	-Positive experiences at school -Negative experiences at school	
		Schools’ awareness of young people’s condition	-Schools awareness	
		Setting educational/vocational goals	-Role of teachers and guidance counsellor (including hospital school teacher) -Coordination between the school and the hospital	
**Theme 4:** Building Support Networks	**Sub-theme 4.1:** Parental and Familial Support	Continued parental involvement	-Parents remaining involved -Parents feel cut out -Parents supporting self-management and development.	This theme highlights the importance of parental and familial support. Parents must provide support to their young people, but also receive support to navigate this challenging transition process.
		Importance of providing parents with support	-Parents teaching themselves to let go -Improving parental support for healthcare transition	
	**Sub-theme 4.2:** Peer to peer support: Utilising formal and informal networks	Importance of renal community	-Peer support -Finding your tribe	This theme emphasises the importance of peer support.
		Relationships with family and friends	-Familial support	
**Theme 5:** Care Coordination, Consistency & Communication	**Sub-theme 5.1:** Care coordination	Lack of coordination	-Absence of coordination -Need for coordination	This theme highlights the importance of care coordination.
		Role of the care coordinator	-Introducing the care coordinator	
	**Sub-theme 5.2:** Consistent care	Importance of routine and consistent staff	-Importance of consistency -Importance of routine	This theme emphasises the importance of routine and consistent staff.
	**Sub-theme 5.3:** Communication	Line of communication to multidisciplinary Healthcare Professionals	-Having a named contact person -Importance of listening -Using appropriate communication strategies	This theme emphasises the importance of communication between families, healthcare professionals and the provision of developmentally appropriate support.
		Evidence of communication between teams	-Multidisciplinary communication	
		Provision of information to young people and their families	-Need for information about healthcare transition -How to provide information -Valuable strategies to make young people more informed.	

The researchers analysed AYA and parent interviews together. During analysis, the research team were cognisant that AYAs and parents may have different experiences and perspectives of HCT. Therefore, while both groups were coded together, codes, categories, and themes were continually compared across groups throughout the analysis, with attention to convergence and divergence between AYAs’ and parents’ accounts. Final themes were retained when they meaningfully represented or significantly contrasted across groups. This process strengthened the analysis, highlighting the person and family-centred nature of HCT, and ensuring that the results were representative of the experiences and perspectives of both the AYAs and parents.

### Rigour & Reflexivity

To maintain methodological rigour, Lincoln and Guba’s criteria for trustworthiness in qualitative research were applied: credibility, dependability, confirmability and transferability [40]. The RTA analysis approach involves continuous reflexivity [39]. This approach embraces researcher subjectivity as an important part of the analysis process [39]. Rather than trying to eliminate the researchers’ influence on the process, RTA encourages researchers to reflect on their positionality and to acknowledge it throughout the research process, particularly when analysing and interpreting data [39]. Credibility and confirmability were enhanced through regular team meetings, during which the researchers critically reflected on their disciplinary backgrounds (nursing and psychology) and on any preconceived ideas or opinions about HCT. The researchers considered how these perspectives may have shaped data collection, analysis, and reporting [40].

For example, the primary researcher remained cognisant of their role as a paediatric nurse. Although the researcher considered themselves an outsider in this research context, with no prior professional relationships with the participants, they had prior knowledge and experience supporting AYAs and families with HCT in a previous professional capacity as a nurse. Continuous reflexivity throughout analysis promoted ongoing reflection and consideration of how this position may have influenced their analysis and interpretation of the results. To further strengthen the credibility of the results, the researcher shared them with participants during the subsequent stage of the study (co-design workshops, which are not reported in this paper) [40].

Dependability was enhanced through maintenance of an audit trail comprising a research diary, memos, and NVivo notes [40]. The primary researcher used these spaces to document all analytical decisions made throughout the study, from data collection and analysis to the reporting of results. The results of the study were derived from participants’ experiences and substantiated by quotes. Transferability was addressed by exploring and providing rich descriptions of the research context, allowing researchers, healthcare professionals and other relevant stakeholders to asses its relevance to other contexts [40].

## 3. Results

Following data analysis, five themes were developed: 1. Complex and ever-changing RRDs, 2. Preparing to move on, 3. Understanding the person, 4. Building support networks, and 5. Coordination, consistency, and communication. To differentiate groups, the following codes were used: AYA (AYAs) and P (parents). Illustrative quotes are presented throughout the results section. See Table 3 for themes, sub-themes and descriptors.

### Theme 1: Walking on a knife-edge: Complex and Ever-changing RRDs

Theme 1 captures participants’ experiences living with complex RRDs, highlighting how these conditions change over time. One participant phrased life with an RRD as “*walking on a knife-edge*” (P-1), emphasising the unpredictable nature of these conditions. This quote captured the essence of this theme. Participants first described their experiences living with RRDs, providing contextual information about their condition. This theme was considered necessary, given that AYAs’ and parents’ HCT experiences are situated relative to their RRD and its impact on their daily lives. The AYAs’ and parents’ experiences living with and managing their RRD are inseparable from their HCT experiences.

For those living with RRDs, HCT experiences are often shaped by the nature and burden of their condition, as well as their ability to cope with and navigate previous or simultaneous life transitions. Therefore, the researchers sought to understand AYAs’ and parents’ experiences living with RRDs. This theme illustrates the complexity and evolving nature of RRDs by focusing on how participants navigate optimism and uncertainty and how their condition shapes their daily lives. These experiences are illustrated across two sub-themes: an uncertain future and the toll of RRDs on everyday life.

### Sub-theme 1.1: An uncertain future: The unknown and the silent

This sub-theme reflects the unpredictability of RRDs, reflecting a sense of an unknown future. Participants shared their experiences living with RRDs, many beginning with diagnosis. While most AYAs had received diagnoses, two were enduring diagnostic tests and procedures. Many of the parents recognised the stress and impact of delayed diagnosis, contributing to uncertainty, treatment delay and disease progression. Those with confirmed diagnoses often continued to face challenges obtaining support due to the rarity of their conditions and difficulty locating expert specialists.

Just one young person reflected on their experience during the diagnostic period. Interestingly, this young person explained how their paediatric clinician had worked tirelessly to confirm their diagnosis. The young person felt that observing their clinician put in such immense effort to provide a confirmed diagnosis ultimately improved their patient-provider relationship and increased their trust in their provider. A few participants reported that,

*“Up until* [Year removed], *I was an undiagnosed condition. They didn’t know why or how I became sick. I was a mystery for a lot of my time in children’s services. But I think what made it so special was* [that] *they never gave up until they found out what it was”* (AYA- 9).

“[Other child] *also had the same condition. So that was a bit of a shock…We kept being told, ‘You’re very lucky’, you know? We had got this far without any major problems. But you know, there were definitely problems looking back.’*” (P-9).

Participants acknowledged the ever-changing nature of complex, multi-system RRDs, demanding specialised care. Many of the AYAs identified that their condition had evolved and was often unpredictable, isolating key transitions, for example, the transition from dialysis to transplantation.

Several AYAs could pinpoint challenging times where they perceived that their condition impacted their life significantly, for example, during periods of relapse, pre-transplantation when on dialysis, the long wait for a matching organ donor, or during the post-transplant recovery period. Many AYAs recalled challenges during ill-health, for example, hospital admissions and constraints imposed by dialysis. Conversely, other AYAs could identify periods where they were well, but still felt somewhat “*different”* (AYA-9) when compared to peers without RRDs or chronic illness. Many AYAs highlighted the unpredictability of RRDs. For example, one young person remained hopeful of receiving a transplant despite the uncertainty. Another had recently experienced disease relapse after several years in remission, emphasising the unpredictable nature of RRDs. According to some participants,

*“I was on the waiting list for* [X] *years, getting a transplant, and I suppose, waking up every day and going to sleep every night you’re waiting for a phone call. You’re waiting for a message.”* (AYA-9).

*“Back in the day, I found it hard because I couldn’t do as much stuff as my friends could…couldn’t really play soccer or anything like that with friends…Then I got my kidney transplant and I have been way* [emphasises] *better.”* (AYA-3).

Comparatively, many parents expressed uncertainty and fear about their child’s future and apprehension about disease relapse or transplant loss or rejection. These parents explained how their children were living with physical complexities, and some reported intellectual disabilities, which often resounded in emotional and sensory challenges. One participant stated that,

*“It is the unknown and the silent. And how quickly it all happened the last time…if you’re not there, not monitoring it, it can be hugely devastating’*” (P-16).

A challenge unique to AYAs with RRDs expressed by one parent was concern about the prospect of becoming a kidney donor, given the unpredictable nature of RRDs and the fear of graft loss. This parent was concerned about becoming a kidney donor and how this would impact upon their ability to support their child throughout the challenging recovery period.

Terms used to describe the unpredictable nature of life with an RRD included “r*oller coaster”* (P-17) and “*the unknown”* (P-16). Some parents described being hyperaware that their AYAs’ condition could deteriorate, prompting urgent medical attention. However, most AYAs reflected a sense of optimism about their condition and did not dwell on the risk of deterioration, despite challenging disease trajectories.

### Sub-theme 1.2: The toll of RRDs on everyday life

This sub-theme reflects the impact of complex RRDs on AYAs’ and parents’ lives. Many of the AYAs interviewed explained that they were in good health, and that their conditions were not affecting their lives to a great extent. However, other young people emphasised physical and psychosocial burden, citing a feeling of difference to peers, challenges with self-identity, frequent clinic visits and complex therapies or medications. These AYAs explained how complex treatment regimens often resulted in challenging side effects, for example, weight gain attributed to steroids, and an inability to participate in sports or other activities alongside peers.

To overcome these challenges, several AYAs reported the need to be organised, to set aside time for medical interventions, and to find new activities that they could safely participate in alongside peers. For example, one young person took up art as a recreational hobby. Another took up golf as an enjoyable, non-contact sport. Nonetheless, other young people had to give up sports that they enjoyed, such as football and rugby, which many found hard to accept, particularly those who had used team-based sports as a social outlet. Some young people reflected on their day-to-day life, remarking,

*“I would describe my day-to-day life, I suppose, as very different to anyone else’s. Ehm, and it can be difficult waking up in the morning, and knowing that I can’t do other things like other people do.”* (AYA-9).

*“If I’m going away, I have to bring those extra supplies. It means, I suppose, even say flying. You have to make sure you have everything that you need for the few weeks on board…it just maybe involves more planning things ahead.*” (AYA-8).

*“I know some people would say ‘Oh, you look great now’. And I’m like, ‘Okay, thanks’. You know. Like, did I not look great like a few weeks ago?...You don’t really care about your appearance or anything like that, I suppose, until you kind of hit teenage years.”* (AYA-11).

Similarly, many parents reported unique challenges, for example, intense treatments with side effects, impacting AYAs’ ability to engage in sports or other activities, contributing further to the physical and psychosocial burden of the condition. Two parents explained that their children had to give up contact sports due to the risk of injury or fatigue. Other parents highlighted the demands on AYAs due to strict fluid or dietary restrictions and interventions, such as urinary testing and enteral nutrition. The parents explained that these tasks often affected family holidays, mealtimes, and child-parent dynamics, especially when AYAs refused to perform condition-specific interventions. Some parents commented that,

*“He would have the lads that he would have palled with. They would have all been big into sport and football and hurling, and whatever, and he reached a point where he had to give up the sports because he couldn’t keep up pace with the rest of the team”* (P-12).

*“He would have had a fluid limit, and his only means of nourishment was via liquid. So that was really tricky.. ehm and created a lot of stress…We couldn’t go to a restaurant, traveling. All of that was really tricky”* (P-7).

*“The older he gets, the harder it is. He is kind of going ‘Ah Mom, I don’t want to. What do I have to do that for?’”* (P-14).

Alongside the physical burden of RRDs, most parents recognised a significant psycho-emotional burden on AYAs’ health. For example, one parent explained that they found their adolescent Googling their life expectancy, feeling that their adolescent “*over obsesses”* (P-16). Another parent recognised how their young person experienced delays receiving treatment, resulting in a life-threatening medical emergency and deep-rooted psychological sequelae, which demanded psychological support that the parent perceived to be not always readily available. This parent described how their young person “*walks on a knife-edge every day”* (P-1). Similarly, many parents recognised a toll on their own well-being, using terminology such as “*it’s very stressful”* (P-6), and “*scaring the living daylights out of me”* (P-16). Others emphasised the condition’s impact on family life, noting its effect on siblings when parents were away.

Some parents described a sense of detachment within families, with parents spending much of their time in the hospital with their young person or attending medical appointments, rather than being together or spending time with siblings. One parent remarked,

*“So* [older child] *was sick, but* [child] *also had a* [sibling] *who was sick…So that was a difficult environment for the* [children] *all growing up, that you know their mommy was away a lot with the younger one.”* (P-9)

While many parents expressed challenging emotions, most AYAs appeared to choose not to allow their condition to dictate their lives, and found ways to overcome and adapt to the constraints attached to their condition. For example, one young person commented that,

*“It* [kidney condition] *can make getting up harder and getting ready, but I have to live with it for the rest of my life, so I can’t let it affect me when I wake up in the morning.”* (AYA-9).

### Theme 2: Preparing to Move on and Managing Change

Theme 2 captures participants’ experiences of preparing to transition into adult services and navigating change. Participants shared their experiences of HCT, which were illuminated across three sub-themes: developmentally appropriate support, empowering AYAs, and anticipating change. Based upon participant accounts, findings suggested that transplant recipients typically attended transition clinics. On the contrary, non-transplanted youth usually did not. Participants described their experiences navigating change.

### Sub-theme 2.1: Age and HCT Readiness

In the context of this study, most AYAs stated that they had, or were due to transition to adult services at sixteen years. Most AYAs felt that this was inappropriate, with many AYAs from paediatric services believing that they lacked knowledge about HCT. Most expressed concern about reduced parental involvement, and concurred that they felt ill-prepared or lacked confidence in their ability to manage their condition independently. Interestingly, two AYAs who had transitioned explained that, although HCT was initially daunting, after the initial adult clinics, their confidence and resilience grew. They adjusted, feeling empowered to navigate their new service.

Others described a sense of limbo, with conflicting emotions about leaving familiar paediatric services versus entering adult services that could cater to their evolving needs. On the one hand, these AYAs expressed a sense of having outgrown paediatric services, as evidenced by being the oldest patients and having outgrown child-centred practices.

These AYAs generally expressed curiosity about adult services and expressed a feeling of being hopeful that the new service would meet their evolving developmental needs. Conversely, they recognised that they would be the youngest in adult services and articulated concerns about changes to routines, staff, and environments. The young people commented that,

*“There is part of me that wants to leave that like.. park it. But there’s also part of me that wants to take it with me”* (AYA-5).

*“I’m honestly gonna miss like being the child… I’m just going to like miss, like, everyone telling my mom. They’ll be telling me instead. But like it’s a nice responsibility to have, but like I feel like I’ll forget everything, so I’ll still need my mom by my side.”* (AYA-7).

Just one AYA who had transitioned in to adult services accepted that HCT was developmentally appropriate at the time. The other AYAs who had transitioned reported feeling ambivalent about leaving paediatric services at the time. Similarly, parents were unanimous that sixteen years was an inappropriate age for HCT, with many expressing frustration that their young people were generally expected to transfer to adult services at 16 years. Several parents recognised that some AYAs with RRDs present with developmental delay, attributed to their condition. Additionally, many explained that AYAs mature significantly between 16 and 18 years, and therefore may be more ready to accept responsibility and manage their condition autonomously at 18 years. Some of the parents noted that,

*“A lot of children that are chronically ill mature very slowly…But then all of a sudden, they’re like ‘Well, they’re sixteen now, so move on’”* (P-5).

*“It is that there isn’t a sharp cut off point. I don’t think there has to be. Now you’re 16 or 18, or whatever. And Mom doesn’t come to the consult anymore. I don’t think that’s how it should be…There has to be like when the river meets the sea type of a situation.”* (P-1).

Several of these parents noted that AYAs can experience difficulty retaining information, self-advocating and managing their condition. While a typical part of growing up, parents emphasised how AYAs require ongoing support and guidance during adolescence, particularly with rare diseases, which have complexities that new providers may not understand. Many parents emphasised that the transfer event does not necessarily indicate transition readiness. Some parents believed that open conversations about HCT early would be helpful. However, many others acknowledged how they felt that AYAs can struggle with change and recommended introducing HCT no more than a year before transitioning, to avoid premature stress. For example, one parent mentioned that,

*“Discussing it* [HCT] *now is going to have it living in his head for the years before he changes, and change is obviously hugely difficult. I’d rather give it a year beforehand, as opposed to three.*” (P-6).

According to participants, the type and level of HCT support provided varied considerably, leading to inequities for those with RRDs. While some AYAs and parents reported receiving preparation for HCT through transition clinics and discussions, many others reported minimal to no preparation. Just two parents felt well-prepared for the transition process. One of these parents had attended an HCT clinic. The other, however, had not. Nonetheless, they had been linked with the adult service in advance of the transition.

The remaining parents reported minimal to no preparation, regardless of whether they had attended a transition clinic. As with parents, there were variations in AYAs’ reports of transition preparation: some perceived they received sufficient information before the transfer event. In contrast, others felt bewildered and isolated after the transfer due to a perception of inadequate preparation. Across participants, attendance at a transition clinic prior to transfer to the paediatric service did not appear to improve AYAs’ or parents’ perceptions of transition readiness. One parent remarked,

*“The preparation was minimal. None. I would describe it as pretty much non-existent.”* (P-2).

### Sub-theme 2.2: Empowering AYAs throughout HCT

This sub-theme reflects how AYAs can be empowered to actively manage their care and advocate for themselves throughout HCT. The AYAs reported mixed decision-making abilities. Most AYAs described a feeling of requiring ongoing support, guidance, and parental input due to a fear of being misunderstood, difficulty understanding complex information, and a perceived lack of confidence in their abilities.

Most AYAs in paediatric services explained that they understood their medications, but stated that their parents continued to support them, for example, reminding them to take medication. For example, some AYAs reported that,

*“I am still very scared. I still always look to my mammy to see what the answer is, even though I know the answer.”* (AYA-9).

*“The small decisions, I think I can make myself. But obviously, the big decisions, I always refer them to my mam.*” (AYA-7).

*“I’ve been in situations with doctors, mostly in adults* [services] *over children’s* [services], *where I found that I’ve been talked over by doctors…when that happens, I get upset because I don’t know how to advocate for myself”* (AYA-4).

Some AYAs who had transitioned into adult services felt independent. Others explained that they appreciated ongoing parental guidance. Those who were independent were generally older and had attended adult services for longer. These AYAs typically had in-depth knowledge of their condition and explained how they valued control over a condition which had greatly restricted other aspects of their lives. For example, one young person stated,

*“I always had a good idea of what my illness was, and the whole medication behind it, but even now... I understand the blood results more, I understand kind of the medication more compared to maybe when I was younger”* (AYA-8).

Similar to the AYAs, the parents expressed concern that their child may not be prepared to receive detailed information about their condition. One parent described how, during their previous clinic appointment, the healthcare professionals had commenced a conversation with their child about their fertility, which their child found distressing. This parent stated,

*“The other thing that came up at the end of the session was about sexual function in the future for* [young person]…*the query of whether* [they would] *be able to* [parent] *a child. And anyway,* [young person] *found it very stressful.”* (P-12).

Additionally, some parents expressed concern that their child did not have a recollection of critical medical events and may not be capable of relaying a complex history to new providers or managing future critical events independently. Some AYAs felt similarly. For example, one young person acknowledged that they required parental support during the initial visits to adult services, to relay their medical history, which they did not have detailed information about. Another parent remarked,

*“He* [son] *wouldn’t have any real recollection of what things were like before he had his transplant. So, in terms of that, as I said, that would be my concern when things do go wrong.”* (P-3).

Strategies deployed by healthcare professionals that many participants found helpful in supporting their transition toward independence and autonomy included encouraging AYAs to answer questions and attend appointments, or components of appointments, independently; gradually introducing new information; and supporting AYAs in performing tasks, for example, filling pillboxes or setting medication reminders. Some AYAs felt that they would have appreciated additional support regarding how to self-advocate and communicate with providers. Some participants expressed,

*“He went into the consultant, spoke to the consultant. I was outside the door…everything was done with* [young person] *leading that. He led that, right down to the follow-up care.”* (P-16).

*“It would definitely be* [beneficial] *to know how to advocate for yourself, and like to have that plan beforehand. I find coming in with like a list on my phone of exactly what I want to say, and even kind of side notes on it, of what I want to happen”* (AYA-4).

For those with intellectual disability, individualised approaches to support AYAs’ independence were considered to be important. Many parents emphasised the importance of ongoing parental or caregiver involvement throughout this cohort’s lifespan. While these parents recognised that their AYAs may never reach complete independence, they emphasised that HCPs should still include AYAs in decision-making and set individualised, appropriate goals. These parents suggested a number of strategies that can be used to support AYAs with cognitive impairment or intellectual disability throughout HCT. These strategies included a person-centred approach, with HCPs taking time to understand the young person, their needs and perspectives, and adopting physical or environmental adaptations, such as sensory or emotional regulation through consistent routines. Involving AYAs in medication management, where appropriate, was also considered important, for example, using pill boxes and tailoring communication strategies, such as plain-language communication during appointments or picture boards.

### Sub-theme 2.3: Navigating change and disrupted expectations

This sub-theme describes how AYAs and parents navigate change while managing accompanying uncertainty and challenges. Most AYAs and parents expressed concerns and uncertainty related to the new adult service environments and processes. Many parents who had yet to transition demonstrated ambivalence due to fear of the unknown, belief that their child was not ready, concerns about adult services, and changing procedures. Most of the AYAs who were yet to transition described feeling concerned about leaving familiar paediatric services, adult environments being less welcoming and loss of parental support. Those who had transitioned articulated their experiences navigating a new service. The AYAs generally acknowledged disparities between services, including feeling like the youngest in the room, longer wait times, and busier environments. These AYAs often described how they struggled with these changes and felt inadequately prepared. Some AYAs stated,

*“It was very upsetting for me, because I just really didn’t want to move at all…I was in child services for* [X] *years, so I knew them people off by heart. I knew their day-to-day routine, so when I went into* [adult hospital] *on the first day, I didn’t know where to go to get my bloods done. I didn’t know where the clinic was… It was very overwhelming.*” (AYA-9).

*“The initial going in, and kind of seeing all the older people. That probably was what stood out to me first, and it kind of leaves you with that question like, ‘Am I the only one this age on dialysis?”* (AYA-8).

One young person explained that they suffered from anxiety, which they felt was exacerbated during HCT due to disparities between expectations and reality. Similarly, most parents who completed HCT described challenging experiences, often resulting in anger and stress, which they perceived to be attributed to fragmented care, absence of a named contact person and a sudden departure from paediatric services. One of the positives articulated by parents was that adult services were often closer to home.

Parents often expressed a feeling that adult services were often ill-equipped for patients with physical and intellectual disabilities, with limited space for wheelchair access, inadequate parking, and a lack of distraction techniques. Additionally, many of the parents recognised changing processes, which initiated concern, for example, procedures in the event of serious illness or when attending emergency departments. One parent remarked,

*“You know you could ring them* [Adult services] *and say, ‘Right, we’re coming in and* [young person] *is really sick’, and you could be four or five hours waiting and even though they knew* [young person] *was on the way, and that* [child] *was very sick. And so we’ve had no good experiences”* (P-13).

Notably, some parents described concern about inpatient stays in mixed adult wards without parental presence overnight. One parent described how their child had a challenging experience when placed in a mixed ward, resulting in trauma and a reluctance to return to the service. Other parents cited longer wait times, which proved especially challenging for patients with intellectual disabilities, and shorter clinics. Similar to AYAs, many parents who had transitioned found switching from the paediatric providers with whom rapport had been established to new adult providers challenging.

Both AYA and parent participants felt that organised visits to adult services in advance of the transition would be beneficial. Interestingly, two parents recognised the potential benefits of remote clinics for routine follow-up appointments, reducing the time spent travelling to the hospital and the impact on their AYAs’ lives, for example, time missed from school. Similarly, most AYAs and parents explained that they would appreciate more support and guidance about what to expect in adult services before the transfer.

Participants noted,

*“Yes, I think it’s hard to know how much* [young person] *is aware of* [their] *surroundings. But you, I suppose, I would have known that it was.. It was new to* [the child]*, and* [child] *was, you know,* [child] *was looking around as well, going, ‘Gosh! This is a small room. How long have I got to sit here for?’ And you know, if I want to get out for ten minutes, where’s the space?”* (P-10).

*“Being able to say like this is the room you go into, this is who you’re going to meet, this is how you’re going to speak to them, would have definitely helped me. Definitely from an anxiety perspective. Because I was going from a very, very familiar situation to something absolutely terrifying.”* (AYA-4).

### Theme 3: Understanding the person and providing individualised and holistic support

This theme describes the need for healthcare professionals to take the time to understand AYAs and to ensure the provision of individualised and holistic support. Most AYAs and parents described the importance of integrating mental health into transitional care support and life integration, i.e., considering other aspects of the young person’s life, such as their sexual, educational or vocational needs. Two quotes which capture the essence of this theme include,

*“Looking at the whole approach rather than just the medical model.”* (P-16).

*“I suppose some healthcare professionals are more aware of.. your life outside. You know you it’s not, ‘Oh, you’ve come to talk about your kidneys. Let’s talk about your kidneys”* (P-10).

### Sub-theme 3.1: Prioritising mental health

This sub-theme emphasises the need to prioritise mental health as a key component of HCT support. The AYAs and parents generally stressed the importance of mental health support and renal counselling, particularly during times of change.

Participants sometimes self-reported mental health disorders, manifesting in anxiety and challenging behaviours, which demanded psychological support that participants perceived to not always be routinely offered. Several parents and AYAs emphasised how, though referrals to mental health services were made, these were often one-off consultations, with no follow-up. Other times, participants described that follow-up was provided, but AYAs found inconsistent staff frustrating. One young person found it challenging to build rapport with mental health professionals, as staff were constantly changing. This young person dropped out of planned therapy sessions due to the continuous need to retell their story. Two young people stated,

*“This is one that really angers me, because there is no one. There is no support group for people like me…My transition clinic in* [Children’s hospital] *lasted, I’d say, seven months, I met one counsellor in them seven months, and I should have probably met one at every appointment.*” (AYA-9).

*“What is the point in like talking to someone* [psychologist], *opening up to someone like, and then they’re just gone? You know? Start the process all over again. Explain the same things again.”* (AYA-11).

Both AYAs and parents emphasised the importance of bridging the gap in mental health support, recognising it as a priority and funding support services. Without this support, AYAs turned to friends or family, which often left AYAs feeling like a burden. The AYAs and parents described that they often sought support from patient organisations, such as the Irish Kidney Association, or from school guidance counsellors, whose support was considered to be invaluable. Others sought private support. Two parents stated,

*“There is a psychologist attached to* [childs] *day services, but the input is very limited. And* [child] *does have challenging behaviours”* (P-7).

*“I never knew that* [young person] *felt a huge responsibility for having the disease and the effect it was having on* [the] *family. So these types of things that came out later should have been coming out earlier”* (P-1).

### Sub-theme 3.2: Two sides of the same coin: Integrating life and HCT

This sub-theme emphasises the need for holistic support that addresses multiple dimensions of AYAs’ lives, not just their medical care. The AYAs and parents recognised simultaneous transitions outside of healthcare, acknowledging that AYAs required support in navigating these aspects of life throughout HCT, for example, sexual health and school. Participants felt that developmentally-appropriate, condition-specific information about sexual health and risky behaviours was important. Just one young person identified that they were routinely asked about sexual health during appointments. The remainder described minimal discussion about sexual health, with most having these conversations at home or at school. These conversations were not condition-specific.

For example, two participants commented,

*“They* [healthcare professionals] *ask you if you have a boyfriend, or if you’re talking to someone. These are all questions that they’re going to ask you all the time. They do talk to you about why you have to tell them, and why it can affect you. And so it is worrying some of the things they tell you. It is worrying. But you have to be sensible growing up, you know?”* (AYA-9).

*“We didn’t really have any of the sexual health from the hospital at all. I just know both* [young people] *with the medicines they are on, that they would need to come off them to have children, so that they have to be extra careful. That would more come from home”* (P-9).

Discussions surrounding risky behaviours, for example, alcohol and tobacco, and their potential harm were common among those who attended transition clinics. The AYAs and parents generally described an awareness of potentially dangerous or risky behaviours, for example, alcohol consumption. Many AYAs described themselves as risk-averse and reported no interest in risky activities. The AYAs from the adult services generally acknowledged that as they got older, they engaged in conversations with HCPs about these topics as they emerged.

Similarly, both AYAs and parents considered educational and vocational support to be an essential aspect of HCT. Many AYAs had positive school experiences and felt well-supported by teachers, special-needs assistants, and guidance counsellors. In these cases, the school staff adapted teaching and assessment strategies, arranged time out of class for frequent toilet breaks, and provided support during absences. One young person reported that,

*“My year head especially...She’s wonderful in what she does, and for me like she really has huge empathy for what I go through sometimes. And yeah… so that’s great. But there’s also some teachers that sometimes can be a bit insensitive”* (AYA-5).

Others described negative school experiences, generally attributed to insufficient knowledge, awareness, and empathy among school staff regarding rare diseases, inadequate adaptation of school activities, non-implementation of school policies (for example, anti-bullying policies), and uniform teaching and assessment strategies that did not support AYAs with additional learning needs. A young person reported that a side-effect of their treatment limited their ability to retain information.This young person explained that the teachers did not initially acknowledge this, which significantly affected the AYAs’ confidence and motivation, leading to a noticeable reduction in their grades and overall school performance.

However, with the support of their hospital psychologist, an intelligence quotient (IQ) assessment was conducted, and the report was shared with the school, heightening the schools awareness and ability to support the young person. Two participants remarked,

*“They* [school] *don’t do anything wrong. It’s just they don’t know what I’m actually doing. Like they know I have kidney failure...but they don’t know like what I am going through.”* (AYA-2).

*“I think the whole myth of ‘the current education system is the only one that works’. I think it’s being open to exploring what’s going to work for that individual.”* (P-16).

### Theme 4: Building support networks

Theme 4 highlights the critical role of support networks, including parents, other family members and peers, in shaping AYAs HCT experiences. Outside of the hospital, participants identified the importance of informal support from parents, family, and peers, as well as more formal support from patient advocacy groups.

### Sub-theme 4.1: Parental and Familial Support

Parents and AYAs emphasised the importance of ongoing support from their families. Most AYAs identified a need for ongoing support from parents and family throughout HCT. Those who had transitioned into adult services generally recognised how, though the level of parental engagement changes as they mature, parents provide emotional support and second opinions. This was particularly important with rare diseases, where adult providers may not be thoroughly familiar with specific heterogenous RRDs. One parent remarked that it is not unusual for a family to accompany adult loved ones to appointments. Yet, they felt that AYAs are frequently expected to attend alone. In addition to supporting their AYAs, many parents reported needing support from healthcare professionals to learn how to safely relinquish responsibility to their AYAs. A young person remarked,

*“I just feel like all the things the doctors say goes in through one ear and then just slips right at the other…I need my mom there…Maybe until I’m 18. Who knows? 20? Forever? I’m not sure but I always need her there.”* (AYA-7).

Many parents recognised the challenge of ‘letting go’ of caregiving responsibilities, particularly for RRDs that require rigid adherence. Many attributed this reluctance to a lack of trust in adult services, often stemming from previous personal experiences. These parents recognised the need for healthcare professionals to support their ability to cope during this change.

Many parents felt that they were not offered support. Those who were offered support described how they had no time to avail of this support due to competing life demands. Two parents commented that,

*“I won’t leave* [child] *alone at all…When* [child] *was on haemodialysis, I never missed a day. Three years in a row, so I’m kind of that person*” [laughs]. (P-11).

*“I knew the support was there if I wanted it or needed it. I just.. It’s like everything else. You knew the crutch was there if you needed it, but I never, I never linked in. I just hadn’t the time.”* (P-17).

### Sub-theme 4.2: Peer-to-peer support: Utilising formal and informal networks

This sub-theme emphasises the importance of peer-to-peer support, through formal and informal networks. All participants discussed the importance of utilising formal and informal peer networks. However, opinions varied regarding the need to mandate peer support. While most AYAs sought support from close friends, some preferred to keep their condition private. Reasons for this included avoiding fuss and unwanted concern and seeking normality. While most AYAs who desired peer support recognised that they did not know others with the same condition, others had met other young people through social media or at the clinic. These young people recognised the value of connecting with peers to share experiences, offer advice, and reduce feelings of isolation. Some participants remarked,

“[AYAs friend] *understands about my sickness and all of that.. like he knows what I can’t eat or what I can eat, or whatever. And he, yeah.. he wouldn’t peer pressure me in to having anything that I am not allowed or anything like that.*” (AYA-3)

*“I don’t know why, but I was very private about it with friends. If my friends were like, ‘where were you yesterday?’, and I just was, you know. I obviously.. I noticed that no one else was really going to hospital, I thought it was like nearly something to be like, not ashamed of, but like I was always like hiding it.”* (AYA-11).

*“I’d say if you said to one of his friends that he was unwell, he’d kill you…They wouldn’t know that he has an issue”* (P-14).

*“It* [Meeting other AYAs] *would be quite refreshing, and sort of it would make me feel much more understood, and I’m sure it would to them as well.*” (AYA-5).

All parents recognised the value of peer support through formal or informal networks. Many parents explained that they had established peer support through online networks, pre-existing advocacy groups, or by meeting others at clinics. Others perceived that they did not have peer support, but desired this support. Several parents emphasised the value of peer networks, explaining how they provide support when navigating complex processes and circumstances, helping to combat isolation. Despite peer support being perceived as predominantly positive, some parents recognised that peer advice should be considered carefully. This was due to prior experiences in which peer discussions heightened their fear or concern about certain events. For example, one parent described how discussions with other parents about HCT had increased their fear and ambivalence toward engaging in the process. Some parents stated,

*“When your child is diagnosed with kidney failure and goes to dialysis, there’s absolutely no other family that you can actually talk to that has gone through that.”* (P-13).

*“It has been really helpful as a mammy, as a parent to have that network. I have met other parents who have kids doing similar things…they are so lovely like, you know they get you.*” (P-5).

### Theme 5: Care Coordination, Consistency & Communication

This theme underscores the importance of the interconnected concepts of consistency, communication and coordination.

### Sub-theme 5.1: Care Coordination

This sub-theme emphasised the importance of care coordination. While not all AYAs explicitly mentioned care coordination, parents interviewed recognised the importance of effective coordination among multidisciplinary healthcare professionals and the need for a designated HCT coordinator. Many parents experienced an absence of coordinated care, leading to negative experiences, including loss of follow-up, missing patient files, and the need to repeat their story. For example, one parent stated,

*“What has since happened is* [young person] *has been dropped by* [specific service]*...*[Young person] *has been dropped in between the cracks.”* (P-16).

Similarly, many parents felt that they had to coordinate their AYAs’ care. This dual role which was perceived to be imposed by systemic gaps in support, meant that they could not just play the role of parent to their young person. Two parents described that they experienced efficient care coordination. In these cases, a healthcare professional assumed the unofficial role of care coordinator in addition to their existing responsibilities, improving service interconnectedness. In both instances, these coordinators arranged multidisciplinary clinics, communicated across teams, and served as a primary point of contact for families. These positive examples, despite being motivated by goodwill, demonstrate the effectiveness and feasibility of care coordinator roles.

Two parents commented,

*“I wanted to be able to be a mom and not have to do all of these other jobs as well.”* (P-1).

*“At the outset, I will say* [Healthcare provider who took on coordinator role] *has been our saviour… she has really coordinated all of the different consultant appointments and everything from the beginning. She was just great at bringing everything under one umbrella. And so that’s, and I think without* [healthcare provider] *it would have been* [laughs] *trickier.”* (P-10).

### Sub-theme 5.2: Consistent care

This sub-theme illustrated the importance of consistent care in RRDs. Both AYAs and parents emphasised the importance of consistency among healthcare professionals, particularly for complex, often multi-system RRDs that require individualised care approaches and specialised knowledge. Both AYAs and parents described the negative consequences of inconsistent staff, including a lack of understanding of rare diseases and their treatment, and the need to repeat their story, which they often found to be distressing. Participants reported,

*“The most scary bit is when you get junior staff…Especially the ones that pretend they’ve heard of the condition before, and they haven’t. I much prefer someone who says ‘I’ve never heard of this’. Then I actually trust that person”* (P-2).

*“When it’s a very rare condition as well…not everybody understands it, so you might be going into a renal clinic with doctors who’ve never heard of this condition…do you know, and don’t really understand it, and they’ll say to you. ‘Oh, but this isn’t what happens’, and you’re saying to them. ‘But this is unusual, because it shouldn’t happen like that’.*” (P-9).

*“You see them* [clinicians] *once, and you never see them again, because they’re not seeing every patient, and they don’t know who I am. They don’t know my case, and it sometimes feels like they’re barely reading my file at all. And they’ve gotten medications wrong, and they’ve gotten things about me wrong.”* (AYA-4).

*“I do find myself explaining my condition a lot. They* [New healthcare professionals] *don’t really understand kind of the ins and out of it.”* (AYA-8).

The AYAs and parents generally believed that consistency and building trusting relationships with healthcare professionals reduced the risk of individuals having to re-explain their condition, which AYAs reported to be fatiguing. Consistent staff were associated with increased safety and trust. One parent emphasised that consistency …was particularly crucial for neurodivergent AYAs. Participants commented,

*“The parent has to trust the person that’s doing the transition…things have got lost, bloods have got lost…So you have to be on top of things for your own child. And so the person that’s doing the transition has to prove their trust to the parents.”* (P-2).

*“The fact that he stays in a hospital on his own and says to me, ‘I’m grown up now you go home’ is a testament to the fact that he feels really safe.”* (P-7).

### Sub-theme 5.3: Communication

This sub-theme captures the importance of effective provider-provider and provider-patient communication. Most participants recognised the importance of clear communication with paediatric and adult providers. Both AYAs and parents recognised that there is a lot of new information and experiences to manage throughout HCT. Therefore, they identified the need for verbal and written information, as well as interactive materials, such as videos, apps, a list of questions to ask healthcare professionals, and welcome packs. Parents of AYAs with impaired cognitive development explained how information should be adapted to their needs, for example, using photos of providers. Several parents emphasised that any resource provided must be accompanied by staff. Without the support of dedicated staff, resources may prove ineffective. Two parents remarked,

*“If there was something that, just like a physical map of the hospital, the cafe, the outpatient clinic, you know, X-ray, the places that you’re most likely to have to go to, just the physical layout of that.”* (P-10).

*“You can have every tool, every piece of paper in the book… You can have every bit of paper, you can tick every box, but it’s all about the people.*” (P-2).

Most participants recognised that during their time in paediatric services, they established strong links with their providers and knew who to contact in emergencies or when concerns arose between appointments. However, upon preparing to transition to adult services, participants described scenarios where information was delivered in an inaccessible format, for example, using jargon that is Not easily understood by those with a non-medical background. Likewise, following the transfer event, many experienced communication barriers, generally attributed to the absence of a named contact person or healthcare professionals lacking awareness of AYAs’ knowledge about their condition and its management. In these cases, the AYAs reported that the healthcare professionals often either undermined or overestimated their knowledge. Participants commented,

*“Trying to make head and tail of what’s being said and abbreviations being used, and not everybody has a medical or healthcare background, and are going in there like a mushroom going, I have no clue what any of this means, or what the implications are. What are you really telling me like? Simplify it in plain English.”* (P-16).

*“There was stuff that they* [Adult clinicians] *would talk about, that I wouldn’t know what’s going on, and there was other times when I would be explained things that were very like basic level of my condition that I knew about. So a discussion, a meeting kind of thing, I think would have been helpful.”* (AYA-4).

Two parents and one young person had positive experiences and felt that communication with their adult providers was efficient. Positive experiences were attributed to the establishment of a strong line of communication with adult providers both before and after the transfer in to adult services, and evidence that paediatric and adult providers were communicating. Many AYAs recognised the importance of adult practitioners communicating effectively with AYAs, through relaxed, open and honest conversations. In general, this improved AYAs’ confidence in providers and willingness to ask questions. Participants stated,

*“I just think a pathway for communication. That you can ring someone or email someone when you’re scratching your head…you’ve come up against a brick wall that you can get someone who you already have a relationship with, who knows all of the players involved, and can see a path out that you’re too confused to be able to see.”* (P-7).

*“I suppose, knowing that you can speak up as well, like, you know, I think for people going through it for so long. And my doctor does understand that if any student comes in and tries talking to me, or, you know, says something, she’s like, ‘No, listen to her.”* (AYA-8).

## 4. Discussion

This study makes a significant contribution to understanding the experiences and unmet care needs of AYAs with RRDs and parents navigating HCT. As one of the first qualitative studies of this unique population, the findings revealed challenges families face when navigating complex, often disjointed health and social systems, the absence of care coordination, and inconsistent HCT practices. These findings highlight and contribute to the call for tailored HCT interventions [5].

### Complex and Ever-changing RRDs: How RRDs shape HCT

Both the AYAs and parents described how living with an unpredictable RRD often meant structuring life around physical and psychosocial challenges, with fluctuating and often unpredictable health. These findings are consistent with the empirical literature, which demonstrates that AYAs with RRDs and their families can have a poorer quality of life than their peers without RRDs [41–44]. These AYAs can experience physical challenges, such as pain, urinary symptoms and fatigue [41], and social and emotional challenges, including ongoing uncertainty, despair and isolation from peers [42,44].

Findings also demonstrated the social burden of RRDs on AYAs lives. For example, impacting their ability to participate in school or other activities. This aligns with the wider literature. For example, Oberdhan et al. [41] reported that 30.3% of AYAs living with polycystic kidney disease who participated in their study experienced challenges participating in sports, and 18.2% experienced challenges engaging in other social activities. In the current study, AYAs generally maintained a positive outlook on life, despite the challenges associated with their condition. Comparatively, parents often expressed concern, attributed to fear about the future, particularly disease relapse or progression. Parental concern about their child’s future is common across rare disease studies [45,46]. Where mismanaged, these concerns can contribute to reluctance to relinquish disease management to AYAs.

### Preparing to Move On

Unsurprisingly, AYAs and parents who perceived that they had received inadequate HCT support were generally less satisfied with their care. This study demonstrated significant inequities in HCT preparation for AYAs with RRDs and the need to standardise services to ensure that they are available to all families, irrespective of their condition. Such inequities are not unique to this study context. Evidence suggests that heterogeneity in HCT practices for AYAs with RRDs is a common phenomenon internationally [47,48], highlighting the need to increase the resources and infrastructure dedicated to HCT for RRDs.

Though various interventions exist internationally to support the HCT process, few have been evaluated in practice [5,49,50]. Additionally, few have been co-designed with families [5]. Participants offered recommendations to improve HCT practices, describing the value of providing opportunities to meet adult providers and visit new services before transitioning, and the potential value of young adult clinics within adult services. These recommendations align with international literature [5,51–53]. The utility of HCT programmes and young adult clinics has demonstrated success in supporting AYAs’ skill acquisition and their ability to navigate adulthood independently [5,51,54].

While recommended as best practice [5,18,48,55,56], any evidence-based programme implemented should be tailored to the needs of AYAs with RRDs and their families [18,57]. This could be managed by individualising HCT care plans, timing the transfer event collaboratively with AYAs and their families, active participation of AYAs and parents in any handovers of care, and the use of patient-specific strategies, for example, picture boards for AYAs with intellectual disability. Another important topic was the interaction between AYAs chronological age and HCT readiness. While age is commonly used to determine the transfer stage, the AYAs and parents in this study concurred that this was inappropriate. The AYAs self-reported mixed perceptions of transition readiness. While some felt that it was a natural milestone, others described a sense of limbo between services. Interestingly, most AYAs from the paediatric services described a feeling of being ill-prepared for transition, largely due to low confidence in their knowledge and abilities and fear about changes in the level of support that they would receive or changing procedures and personnel in adult services.

Parents felt similarly and were generally concerned about AYAs’ ability to manage their condition independently. Multiple studies support this conclusion [49,55,56]. For example, a study by van Staa et al. [52] revealed that 56% of AYAs felt ready for transfer, with self-efficacy and independence being more critical factors than age.

Likewise, a study by Rutishauser et al. [58] noted that anxiety and lack of information are significant barriers to transition. In Rutishauser et al. [58] study, participants preferred to transfer to adult services between 18 and 19 years (50%), 16-17 years (30%) and 20 years plus (14%) [58]. In their study, Rutishauser et al. found that the age preference for AYAs with chronic illness and their parents was in fact greater than the age limit for paediatric services across many European countries [58]. Tournivuori et al. [59] proposed that transition readiness should be assessed based upon AYAs disease knowledge, self-management, and psychosocial skills. Findings from this study indicate that age alone is not a reliable measure of transition readiness, and age does not reflect an AYA’s ability to manage their healthcare independently.

Ongoing fluctuations and variability in AYAs’ cognitive, emotional, and social maturation demand individualised approaches to assessing transition readiness and tailoring support. Prematurely transitioning AYAs may contribute to poor outcomes. Therefore, applying inflexible age restrictions without acknowledging AYAs’ maturation or individual HCT readiness may conflict with the importance of individualising care [47] and, for AYAs with RRDs, can prove detrimental. This study reinforced that HCT is more than a service-level transfer; rather, it is about holistically supporting AYAs’ transition from childhood to adulthood. Therefore, calling for flexibility surrounding the specific timing of the transfer event, and recognition of HCT as a process that continues into adult services following this event.

### Building Support Networks

The important supportive roles of parents and peers were emphasised. Parents explained how delegating the care of complex, life-threatening RRDs to AYAs who may not be prepared for this responsibility could prove hazardous. Additionally, parents faced challenges entrusting care to new providers, who often had limited knowledge about RRDs. Research consistently demonstrates the challenges that parents face when living with a child with an RRD [46,57]. Challenges include a struggle to balance protecting their child with supporting their transition towards independence and autonomy [49,60]. Shaw et al. [61] described the need for healthcare professionals to support parents by equipping them with knowledge and skills to support their AYAs during this critical period and to help them to relinquish control to their young person. As described by Shaw et al. [61], “A confident parent breeds a confident child” (p. 312). Co-creating individualised approaches to support parents throughout HCT may be valuable.

The AYAs in this study appreciated their parents’ ongoing support throughout the HCT process. This sentiment applied to both those in paediatric services and those who had transitioned. While the AYAs acknowledged that parents roles and the level of parental support change, they generally still valued parental engagement in their healthcare. This aligns with findings from Badour et al.’s review. [62], which demonstrated that maintaining parental engagement throughout HCT generally has a positive impact on HCT outcomes. However, there is a tenuous balance between parental support and micromanagement [5,62]. Therefore, parental engagement should be considered on an individual basis, with the decision made collaboratively with AYAs, parents and HCPs.

Participants maintained strong support networks through family and peers, though some AYAs concealed their condition from friends. Reasons for this included a desire for normalcy or an attempt to evade stigmatisation and bullying. This aligns with a recent study by Somanadhan et al. [63], which found that children living with rare diseases frequently report experiencing stigmatisation and self-consciousness. Evidence suggests a link between a desire for normalcy and disease avoidance with non-adherence to treatment [64], contributing to poor health outcomes. Research consistently demonstrates the value of peer support throughout HCT [5,61]. Peer support may promote a sense of empowerment, belonging and shared social identity [65–67].

While many of the AYAs and parents interviewed emphasised the value of peer support, some parents whose AYAs did not participate in the study recognised that they did not expect that their young person would engage in such peer support groups. Therefore, peer support may not be for everyone and should be considered optional. Likewise, parents acknowledged potential risks associated with peer support, including misinformation and unintentional scaremongering, which corresponds with the literature [68]. To overcome these risks, Joo et al. [69] recommended training and service supervision of designated peer supporters.

### Understanding the Person

Parents and AYAs emphasised the need for psychological support, particularly when navigating challenging life transitions, particularly health transitions. Evidence suggests that AYAs with RRDs are at increased risk of mental health disorders [42,70]. Psychological support has been cited as a top priority [70,71], and should be considered particularly important during HCT. In the present study, AYAs described feeling isolated, dejected and uncertain at this junction in their healthcare journey. One parent noted that their young person had revealed that they felt like a burden. Similarly, parents reported stress, ambivalence and worry.

Despite psychological and emotional turmoil, participants reported a perceived lack of or inconsistent psychological support. The need to prioritise genetic counselling and mental health support was emphasised. Anxiety and a lack of information about HCT were among the most critical barriers for a smooth and timely transfer, according to AYAs and parents.

Socially, participants emphasised the importance of recognising aspects of their lives outside of the hospital and of improving the interconnectedness between health and social systems, particularly schools. While some AYAs had positive school experiences, many faced challenges. National and international HCT recommendations suggest that HCPs set academic and vocational goals with AYAs [12]. However, participants did not perceive this as beneficial. Both AYAs and parents perceived teachers and guidance counsellors at school to be the most appropriate people to provide this support. Nonetheless, the AYAs and parents desired improved collaboration between health services and schools. Recommendations included directing AYAs to disability supports and conducting assessments (for example, screening for additional learning needs), so that school staff can adapt teaching and assessment strategies and implement policies (for example, anti-bullying) to support AYAs.

### Care Coordination, Consistency and Communication

The importance of coordination, consistent care and effective communication was highlighted. The rare, multi-system nature of RRDs can make care coordination challenging, requiring AYAs to attend multiple specialists and appointments, often on different days or in different health service institutions [72,73]. Parents emphasised the value of HCT coordinators in both paediatric and adult services. Despite national and international advocacy for HCT coordinator roles [47], only two parents were supported by care coordinators. These parents explained that these were not official roles; instead, HCPs assumed the role selflessly. Both of these parents emphasised that, without this support, HCT would have been problematic, given that their AYAs had complex multisystem needs spanning multiple disciplines and, in one case, across different services.

In particular, HCT coordinators have demonstrated success across other contexts. Lemke et al. [74] found that care-coordinator roles improved families’ experiences. Similarly, Sandquist et al. [75] found that system-based care coordinator roles were the most effective HCT intervention for rare diseases. Despite their value, the practice remains inconsistent. Kreuzer et al. [47] reported that less than two-thirds of the European centres of expertise for RRDs have designated HCT coordinators. Moreover, participants emphasised the importance of provider-to-provider, provider-to-patient, and provider-to-parent communication. Despite communication being a crucial element of care coordination [73], most participants reported poor communication practices. Some reported missing files and absent handovers of clinical information between providers. Providing opportunities for paediatric and adult providers to meet families through joint clinics may be helpful. Additionally, electronic health records may improve information sharing. The AYAs and parents reported that HCPs should enhance their communication skills, use non-technical language, and take time to build rapport with AYAs and parents.

Finally, the importance of consistent care in the context of rare diseases was emphasised. Inconsistent staff was associated with fragmented care, increased risk of adverse events, psychological stress and lack of trust. This aligns with a previous national study by Somanadhan et al. [46], which found that nearly half of the rare disease parents included in their study reported inconsistent healthcare professionals for their children, negatively impacting communication and care coordination.

### Strengths & Limitations

This study is among the first to examine HCT from the perspective of families with RRDs. The RTA approach was employed to promote researcher reflexivity in the collection, analysis, and presentation of findings, thereby enhancing the study’s rigour. The study captured diverse perspectives of various RRDs and groups, including those receiving RRTs, undiagnosed groups and those with complex needs. The samples’ diversity may be attributed to collaboration with national paediatric services, RD organisations and patient advocacy groups.

Some limitations must also be considered. Methodologically, the study sample included 17 parents and 11 AYAs. While qualitative studies do not always strive for an even distribution of participants from different groups, but rather focus on reaching information power [28], this imbalance may reflect parents who shared their HCT experiences on behalf of AYAs with intellectual disability who could not participate. The imbalance may also be related to recruitment challenges with young adults. The research team remained cognisant that higher parental representation may have resulted in possible parental anxiety and over-reporting, influencing the conclusion of this study. To mitigate this challenge, several measures were implemented to ensure the rigour and credibility of findings were upheld. Firstly, while more parents were interviewed, information power was reached for both groups.

Similarly, the analysis approach taken accounted for the imbalance between AYAs and parents. As discussed in the methods section, themes were retained when they represented the opinions of both groups or demonstrated significant divergence. While this study provides valuable insights, future studies are recommended to explore the transferability of these findings in other contexts.

An additional limitation to consider was that of the 28 interviews conducted, only 4 were in person. The remaining interviews were conducted *via* Zoom. Participants were given the option of attending *via* Zoom or in person for their convenience, comfort and to promote engagement from participants who lived remotely. Both online and in-person interview formats have strengths and limitations [76]. While the higher number of Zoom interviews reflected participants’ preferences, it may have introduced variation in the interview dynamics. To mitigate these potential challenges, the researcher used the same interview structure and guide across all interviews and took reflexive notes throughout. Similarly, all participants who opted for Zoom kept their cameras on to promote rapport building [76].

Additionally, time was taken at the start of both the online and in-person interviews to build rapport with participants. While reflecting, the researcher considered variations in responses from those who participated online compared to those who participated in in-person interviews. However, no noticeable differences were observed in the level of interaction across the sessions.

An additional challenge faced by the researcher was that although every effort was made to secure representation from fathers, the researchers were able to recruit only one father. This is consistent with international literature, demonstrating that mothers are more often primary caregivers to children with rare diseases or complex needs [77]. Nonetheless, this highlights the need for targeted, father-specific recruitment approaches in future studies. For example, including fathers explicitly in recruitment messaging on research posters. Finally, while factors such as gender and diagnoses may shape HCT experiences, this study did not report these characteristics. This decision was made to honour ethical obligations to protect participants’ anonymity, given the heterogeneity of RRDs and the potential for reidentification. This decision was considered appropriate given that these demographic details were not central to the analysis.

## 6. Conclusion

Healthcare transition is a holistic process that does not end with the transfer of care. This study highlights the need for coordination between providers across specialities, effective communication and consistent care. This study emphasises the dynamic interplay between health and social systems when planning HCT. Sustainable, practical HCT interventions must be implemented to meet the needs of this unique cohort.

## Supporting information

S1 File COREQ Checklist. (PDF)

S2 File Interview Guides [Parents completed/preparing for HCT & Young people completed/preparing for HCT]. (DOCX)
