# Supplementary material for: The river meeting the sea: A qualitative exploration of the healthcare transition experiences of adolescents and young adults living with rare renal disorders and their parents
Source: PLoS One. 2026 Jun 3;21(6):e0348445. doi: 10.1371/journal.pone.0348445 (PMC13232946; doi:10.1371/journal.pone.0348445)
Supplement: S2 File — (DOCX) [file pone.0348445.s002.docx]

**Interview Guide (Parents/Caregivers who have completed transition)**

1. **Introduction**
2. The interviewer (MK) introduces herself.
3. The purpose of the interview is stated.
4. Duration: 40-60 minutes (60 minutes maximum).
5. **Interview**
6. The interview opens with general questions, such as asking the participant:
7. How old is your child?
8. When did your child move to adult services?
9. What is your child’s diagnosis?
10. How would you describe you and your child’s day-to-day life living with the diagnosis/How does this diagnosis impact their day-to-day life?

**Interviews:** Today’s discussions focus on the movement between child and adult services for young people, like *insert name*, who are living with a kidney condition and their families.

**Topic One: Current transition clinic experience**

- How did you experience the movement between child and adult services?
- What can you tell me about the preparation you and your child received for moving to adult services? What were your experiences? (*This question may require additional prompts*).
- Did anyone support you and your child’s move between child and adult services? *Prompt: If so who? Can you tell me more about this support?*
- How did you feel about this move?
- If you could make changes to the current preparation for the movement between child and adult services, if any, what would these changes be? (*Prompt: Was there anything particularly helpful/unhelpful?*).
- Tell me, was there anything in particular that you found useful during the preparation period?
- What mattered most to you at this point? What were your needs? Were these needs met?

**Topic Two: Self-Advocacy and Independent Healthcare Behaviour**

- Does someone support your young person with their care? (*Examples may need to be provided*).
- Tell me, does your young person understand their medications, when and why they take them? In what ways do they demonstrate this knowledge?
- Does someone support your child with medication management?
- Did you think that your child was ready to make decisions about their care, for example, booking appointments, when they moved to the adult services? What about your child? Can you expand on this further?
- What is your thought about how your child was prepared to make decisions independently during the move to adult services?
- Do you feel that they had a good understanding of their condition before moving into adult services? *Prompt: If so, how was this demonstrate or supported? If not, how could they have been better supported?*
- How did you feel about your young person attending the clinic alone? / Do you feel that your young person was ready to attend the clinic alone? Tell me more about this.
- Can you describe a time that your young person were involved in decisions about their condition and its management before moving to adult services? If so, how was this demonstrated? If not, how could this be better supported?
- How did you find the move to adult services?

**Topic Three: Sexual Health and potential risks**

- What kind of discussions, if anything, pre-transfer did your young person have surrounding sexual health before moving to adult services?
- Were you and your child aware of high-risk behaviours and how to avoid them before moving to adult services? What high-risk behaviours were you made aware of? Who made you aware of these risks? (*Examples may need to be provided, for example, drinking alcohol or smoking tobacco*).

**Topic Four: Psychosocial Support**

- Tell me, who supports you and your child at home, outside of the hospital?
- Are you well supported as a parent? Can you tell me more about this?
- What kind of support is available to both you and your child within and external to the hospital? For example, what about in school or at work, if you have a job?
- What else, if anything, could healthcare professionals do to support you and your child?

**Topic Five: Education and Vocational Planning**

- Tell me, as part of the preparation for the move to the adult hospital, did you and your child have the opportunity to discuss and plan your life with someone and set goals? Prompts: If so, who supported you? How did you find this?
- If not, is this something that you would have found valuable?
- If you did, what sort of goals did you plan?
- Is there any other support you would find helpful?

**Topic Six: Interventions**

We hope to develop supportive resources for young people and their families with the move to adult services.

- If you had a magic wand and could improve how healthcare transition is delivered, what kind of resources do you not currently have but wish were available?
- If you could design a resource (SOMETHING), what would it look like? (*Prompt: This could be anything, from an app for your phone to a youth camp*).
- Is there anything else that you would find helpful?
- What is the most valuable piece of advice that you would give to other young people and/or parents/caregivers who are moving to adult services?
- Are there any conversations you wish you had with your paediatric provider before moving to adult services?

**Conclusion**

- Is there anything else you would like to discuss or mention that we have not discussed today?

**Interview Guide (Parents/Caregivers who are preparing for transition)**

1. **Introduction**
2. The interviewer (MK) introduces herself.
3. The purpose of the interview is stated.
4. Duration: 40-60 minutes (60 minutes maximum).
5. **Interview**
6. The interview opens with general questions, such as asking the participant:
7. How old is your child?
8. When will your child move to adult services?
9. What is your child's diagnosis?
10. How would you describe you and your child's day-to-day life living with the diagnosis/How does the diagnosis impact their day-to-day life?

**Interviews:** Today’s discussions focus on the movement between child and adult services for young people, like *insert name*, who are living with a kidney condition and their families.

**Topic One: Current transition clinic experience**

Do you know anything/have you been told anything about the movement between child and adult services? *Prompt: What do you know?*

- Are you and your child receiving any support at the moment to prepare for the move to adult services? What are your experiences? (*This question may require additional prompts*).
- Does anyone support you and your child with the move between child and adult services? *Prompt: If so,* *Who? Can you tell me more about this support?*
- How do you feel about this move?
- If you could make changes to the current preparation for the movement between child and adult services, if any, what would these changes be? (*Prompt: Was there anything particularly helpful/unhelpful?*).
- Tell me, what do you find useful during the preparation period?
- What matters most to you at this point? What are your needs? Are these needs met?

**Topic Two: Self-Advocacy and Independent Healthcare Behaviour**

- Does someone support you and your child with their care? (*Examples may need to be provided*).
- Tell me what do you think is your child’s understanding of their medications, including when and why they take them?
- Does someone support your child with their medication management?
- Tell me, what you think about your child’s preparation to make decisions about their care, for example, booking appointments, when they move to the adult services? How do you think this preparation could be improved?
- Tell me, do you feel they have a good understanding of their condition? How so? If not, how can this be better supported?
- How do you feel about your child attending the clinic alone? /Do you feel that they are ready to attend the clinic alone? Can you expand on this?
- Has your child been able to make decisions about their condition and its management before moving to adult services? If so, how was this demonstrated? If not, how could this be better supported?

**Topic Three: Sexual Health and risky behaviour**

- Have the healthcare team discussed sexual health with you and your young person as part of the clinic? If so, what did they discuss?
- Are you and your child aware of high-risk behaviours and how to avoid them before moving to adult services? What are these risks and how do you know about them? (*Examples may need to be provided, for example, consuming alcohol or smoking tobacco*).

**Topic Four: Psychosocial Support**

- Tell me, who supports you and your child at home, outside of the hospital?
- What do you think about the level of support that you have, as parent?
- What kind of support is available to both you and your child within and external to the hospital? For example, what about in school or at work, if you have a job?
- What else, if anything, could healthcare professionals do to support you and your child?

**Topic Five: Education and Vocational Planning**

- Tell me, as part of the preparation for the move to the adult hospital, have you and your child had the opportunity to discuss and plan your life with your social worker and to set goals? If so, how did you find this?
- If not, is this something that you would find valuable? Why? Why not?
- What sort of goals did you plan?
- Is there any other support you would find helpful?

**Topic Six: Interventions**

We hope to develop supportive resources for young people and their families with the move to adult services.

- If you had a magic wand and could improve how healthcare transition is delivered, what kind of resources do you not currently have but wish was available?
- If you could design a resource (SOMETHING), what would it look like? (*Prompt: This could be anything, from an app for your phone to a youth camp*).
- Is there anything else that you would find helpful?
- What is the most valuable piece of advice that you would give to other young people and parents/caregivers who are moving to adult services?
- Are there any conversations that you would like to have with your healthcare provider that you have not yet had that would help you prepare for the move to adult services?

**Conclusion**

- Is there anything else you would like to talk about or mention that we have not talked about today?

**Interview Guide (Young People who are preparing to transition)**

1. **Introduction**
2. The interviewer (MK) introduces herself.
3. The purpose of the interview is stated.
4. Duration: 40-60 minutes (60 minutes maximum).

**B. Icebreaker**

1. Tell me about your favourite film?
2. What do you like to do in your spare time?

**C. Interview**

1. The interview opens with general questions, such as asking the participant:
2. How old are you?
3. How long have you been attending the hospital?
4. What is your diagnosis?
5. How would you describe your day-to-day life living with the diagnosis/How does the diagnosis impact their day-to-day life?

**Interviews:** Today’s discussions have a specific focus on the movement between child and adult services for young people like you, who are living with a kidney condition.

**Topic One: Current transition clinic experience**

- Has anyone discussed or told you anything about the movement between child and adult services? *Prompt: What have you been told? Who has chatted with you about this?*
- How do you perceive the preparation for the move to adult services? What are your experiences of healthcare transition? (*This question may require additional prompts*).
- Is anyone supporting you with the move between child and adult services? *Prompt: If yes, what sort of support have you received?*
- Can you tell me about how you feel about this move?
- If you could make changes to the current preparation for the movement between child and adult services, if any, what would these changes be?
- Tell me, have you found anything helpful during the preparation period? *Prompt: Can you tell me more about this?*
- What matters most to you currently? What are your needs? Are these needs met?

**Topic Two: Self-Advocacy and Independent Healthcare Behaviour**

- Does anyone support you with your care? (*Examples may need to be provided*).
- What is your level of understanding about your medications, including when and why you take them?
- Does anyone support you with managing your medications? *Prompt: Can you tell me more about this?*
- Do you feel ready to make decisions about your care, for example, booking app.? Tell me more about this.
- Tell me about your understanding of your condition?
- How do you feel about attending the clinic alone?
- Have you been able to make decisions about your condition and its management? What kind of decisions have you been able to make?

**Topic Three: Sexual Health and risky behaviour**

- Have the healthcare team discussed sexual health with you as part of the clinic? If so, what did they discuss?
- Do you know about any high-risk behaviours that are associated with your disease and how do you avoid them? How do you know about these risks? (*Examples may need to be provided, for example alcohol consumption or smoking tobacco*).

**Topic Four: Psychosocial Support**

- Tell me, does anyone support you at home, outside of the hospital?
- What do you think about the level of support that you have?
- Is there any support available to you both within and external to the hospital? *For example,* what about in school or at work, if you have a part-time job?
- Are you aware of any mental health supports or social supports (people that you can talk to) that are available for you? If so, can you name them?
- What else, if anything, can healthcare professionals or other people, for example, teachers, do to support you?

**Topic Five: Education and Vocational Planning**

- Tell me, as part of the preparation for the move to the adult hospital, have you had the opportunity to discuss and plan your life with anyone, and to set goals? -If so, how did you find this? -If not, is this something that you would find valuable?
- What sort of goals did you plan?
- Is there any other support you would find helpful?

**Topic Six: Interventions**

We hope to develop supportive resources for young people like you and their families with the move to adult services.

- If you had a magic wand and could improve how healthcare transition is delivered, what kind of resources (or things) do you not currently have but wish were available?
- If you could design a resource, what would it look like? (*Prompt: This could be anything, from an app for your phone to a youth camp*).
- Is there anything else that you would find helpful?
- What is the most valuable piece of advice that you would give to other young people and parents/caregivers who are moving to adult services?
- Are there any conversations you would like to have with your healthcare professional that you have not already had?

**Conclusion**

- Is there anything else you would like to discuss or mention that we have not discussed today?

**Interview Guide (Young People who have completed healthcare transition)**

1. **Introduction**
2. The interviewer (MK) introduces herself.
3. The research objectives and interview objectives are stated.
4. The interviewer discusses the semi-structured style.
5. Duration: 40-60 minutes (60 minutes maximum).

**B. Icebreaker**

1. Tell me about your favourite film?
2. What do you like to do in your spare time?

**C. Interview**

1. The interview opens with general questions, such as asking the participant:
2. How old are you?
3. How long have you been attending the adult hospital?
4. What is your diagnosis?
5. How would you describe your day-to-day life living with the diagnosis?

**Interviews:** Today’s discussions focus on the movement between child and adult services for young people like you who are living with a kidney condition.

**Topic One: Current transition clinic experience**

- How did you experience the transition between child and adult services?
- What can you tell me about the preparation you received for moving to adult services? What were your experiences of healthcare transition? (*This question may require additional prompts*).
- Did anyone support you with the move between child and adult services? *Prompt: If yes, what sort of support did you receive?*
- Tell me, how did you feel about this move?
- If you could change the current preparation for the movement between child and adult services for other young people, if anything, what would these changes be and why?
- Tell me, did you find anything useful during the preparation period? *Prompt: Can you tell me more about this?*
- What mattered most to you at this time? What were your needs? Were these needs met?

**Topic Two: Self-Advocacy and Independent Healthcare Behaviour**

- Does anyone support you with your care? (*Examples may need to be provided*).
- Tell me, what is your level of understanding about your medications, including when and why you take them?
- Does anyone support you with managing your medications? *Prompt: Can you tell me more about this?*
- How do you feel about making decisions about your care? When you moved to adult services, how did you perceive the level of preparation you received to make your own decisions about your care and self-manage your condition etc.?
- Tell me about your understanding of your condition?
- Tell me about your first appointment in the adult services. How did you feel? Do you feel that you were prepared for your first clinic appointment in the adult hospital? Can you elaborate on this?
- How did you feel about attending the appointment alone? Were you prepared for this?

**Topic Three: Sexual Health and Risky Behaviours**

- Can you remember, did anyone discuss sexual health with you as part of the paediatric clinic before you moved to adult services? If so, what did they discuss?
- Were you aware of any high-risk behaviours and how to avoid them? What are these risks and how did you know about them? (*Examples may need to be provided, such as consuming alcohol or smoking tobacco*).

**Topic Four: Psychosocial Support**

- Tell me, does anyone support you at home, outside of the hospital?
- What do you think about the level of support that you have?
- Is there any support available to you both within and external to the hospital? For example, what about in school or at work, if you have a part-time job?
- Are you aware of any mental health or social supports (people that you can talk to) that are available for you? If so, can you name them?
- What else could healthcare professionals do to support other young people like you?

**Topic Five: Education and Vocational Planning**

- Tell me, as part of the preparation for the move to the adult hospital, did you have the opportunity to discuss and plan your life with anyone and to set goals? If so, how did you find this?
- If not, is this something that you would find valuable?
- What sort of goals did you plan?
- Is there any other support you would have found helpful?

**Topic Six: Interventions**

We hope to develop resources for young people and families.

- If you had a magic wand and could improve how healthcare transition is delivered, what kind of resources do you not currently have but wish were available?
- If you could design a resource (or thing), what would it look like? (*Prompt: This could be anything, for example, from an app for your phone to a youth camp. It could also be something easy, like a transition passport*).
- Is there anything else that you would find helpful?
- What is the most valuable piece of advice that you would give to other young people and parents/caregivers who are moving to adult services?
- Are there any conversations you did not have with your healthcare team in the child setting that you wish you had?

**Conclusion**

- Is there anything else you would like to discuss or mention that we have not discussed today?
